# Early Life Disruption of the Microbiota Affects Organ Development and Cytokine Gene Expression in Threespine Stickleback

**DOI:** 10.1093/icb/icaa136

**Published:** 2020-09-24

**Authors:** Lucas J Kirschman, Anastasia Khadjinova, Kelly Ireland, Kathryn C Milligan-Myhre

**Affiliations:** Department Biology, Southeast Missouri University, 1 University Plaza, Cape Girardeau, MO 63701, USA; School of Medicine, University of Washington, Seattle, WA 98195, USA; Department of Biological Sciences, University of Alaska Anchorage, Anchorage, AK 99508, USA; Department of Biological Sciences, University of Alaska Anchorage, Anchorage, AK 99508, USA; Department of Molecular and Cell Biology, University of Connecticut, Storrs, CT 06269-3125, USA

## Abstract

The microbiota that inhabits vertebrates exerts strong effects on host physiology and can be crucial to the development of a normal phenotype. This includes development of the immune system, somatic growth and maintenance, and morphogenesis. However, the genetic background of the host can also affect these life history traits. To this end, we investigated the effects of the microbiota on growth, development, and immune gene expression on two populations of threespine stickleback (*Gasterosteus aculeatus*), one anadromous and one freshwater. We tested the hypotheses that microbial colonization and the genetic background of the host would affect survival, cytokine gene expression, growth, and development. We raised *in vitro* crosses of stickleback larvae with and without conventional microbiota. We then exposed all these treatments to *Vibrio anguillarum*, a potential fish pathogen, in a full factorial design. We found stickleback raised without conventional microbiota had smaller swim bladders relative to those raised with conventional microbiota. Stickleback raised with conventional microbiota exhibited small increases in cytokine gene expression. We found no differences in growth or survival regardless of treatment. These results are consistent with other investigations that show microbiota disruption, in early life, can alter host organ and tissue development and immune responses

## Introduction

The microbes that colonize and inhabit vertebrates can exert strong effects on host phenotype and physiology (Clemente et al. 2012; McFall-Ngai et al. 2013; Bordenstein and Theis 2015; Zaneveld et al. 2017). However, there appear to be critical windows, particularly in early life, in which a healthy microbiota is essential for the development of a normal phenotype (Sommer and Bäckhed 2013; Arrieta et al. 2014; Borre et al. 2014; Cox et al. 2014; Warne et al. 2019). This includes the maintenance of somatic growth, which demands a substantial amount of energy in immature animals (Pedersen 1997; Van Leeuwen et al. 2012; Rosenfeld et al. 2015). The microbiota aids the host in the acquisition of energy and nutrients and can thus exert large effects on the growth rate of immature animals (Pryor and Bjorndal 2005; Schwarzer et al. 2018; Warne et al. 2019).

The microbiota also affects the development of many organ systems, including the immune system. For example, in order to sequester and interface with the microbiota, most clades of jawed vertebrates have evolved gut-associated lymphoid tissue and immunoglobulins specific to the mucosal immune system (Brandtzaeg 2009; Zhang et al. 2010; Rombout Jan et al. 2011; Hooper et al. 2012; Kaetzel 2014; Pettinello and Dooley 2014; McCoy et al. 2017). However, microbial colonization is essential for the proper development of both the mucosal and systemic immune systems (Kanther and Rawls 2010; Hooper et al. 2012; Sommer and Bäckhed 2013; Gensollen et al. 2016; Thaiss et al. 2016; McCoy et al. 2017). Disruption of the microbiota during early-life development in animals is associated with reduced immune function and increased disease susceptibility (Abt et al. 2012; Arrieta et al. 2014; Honda and Littman 2016; Warne et al. 2019). Furthermore, the healthy microbiota can bolster immune function by outcompeting enteric pathogens for niche space (Gómez and Balcázar 2008; Arrieta et al. 2014; Thaiss et al. 2016). In addition to the immune system, the early-life microbiota also exerts effects on the morphogenesis and physiology of the gastrointestinal tract (Sommer and Bäckhed 2013; Troll et al. 2018), metabolic rate (Cox et al. 2014), and brain development (Sampson and Mazmanian 2015).

Here, we investigated the effects of the microbiota on growth, development, and immune function during early life. Animals in early-life stages often exhibit negative associations between immune responses, development, and somatic growth due to energetic trade-offs (Arendt 1997; Van Der Most et al. 2011; Kirschman et al. 2017, 2018). There is evidence that the microbiota may affect all three of these traits by increasing energy acquisition, influencing neuroendocrine signaling, or upregulating immune function (Warne et al. 2019). Furthermore, while effective immune responses during early-life stages can prevent pathogens from impairing host growth or development, the immune responses themselves may result in similar impairments (Shanks et al. 2000; Newburg and Walker 2007; Petri et al. 2008; Strunk et al. 2011; Dowling and Levy 2014).

Somatic growth rate, development, and immune function are also products of host genetic background. To this end, we used threespine stickleback (*Gasterosteus aculeatus;* stickleback), a biomedical model organism, to determine how the host genetic background influences the ability of the microbiota to affect development. Stickleback have been used for over a century to elucidate the effects of the environment on evolution (Kimmel et al. 2005; Ostlund-Nilsson et al. 2006; Miller et al. 2007). Multiple populations of freshwater stickleback have evolved from common, anadromous ancestors in habitats with distinct chemical and biotic characteristics. Thus, they exhibit interpopulation genetic variation that affects host–microbe interactions. Genetic distance between stickleback populations explains more of the variance in their microbiota community structure than their physical environment or geographical distance from one another (Smith et al. 2015; Steury et al. 2019).

We previously found that the divergent genetics of stickleback populations seem to regulate the host response to the microbiota. For example, anadromous stickleback exhibit greater inflammatory responses to common microbes than freshwater populations (Milligan-Myhre et al. 2016; Small et al. 2017). These differences in host immune responses reflect other studies that have shown differences in immune responses between stickleback populations (Kurtz et al. 2004; Scharsack and Kalbe 2014; Weber et al. 2017). While these populations vary in the development of specific bone morphology (Currey et al. 2017), to our knowledge, there are no studies examining how microbial colonization in early life affects somatic growth, development, and immune responses between different stickleback populations.

**Fig. 1:**
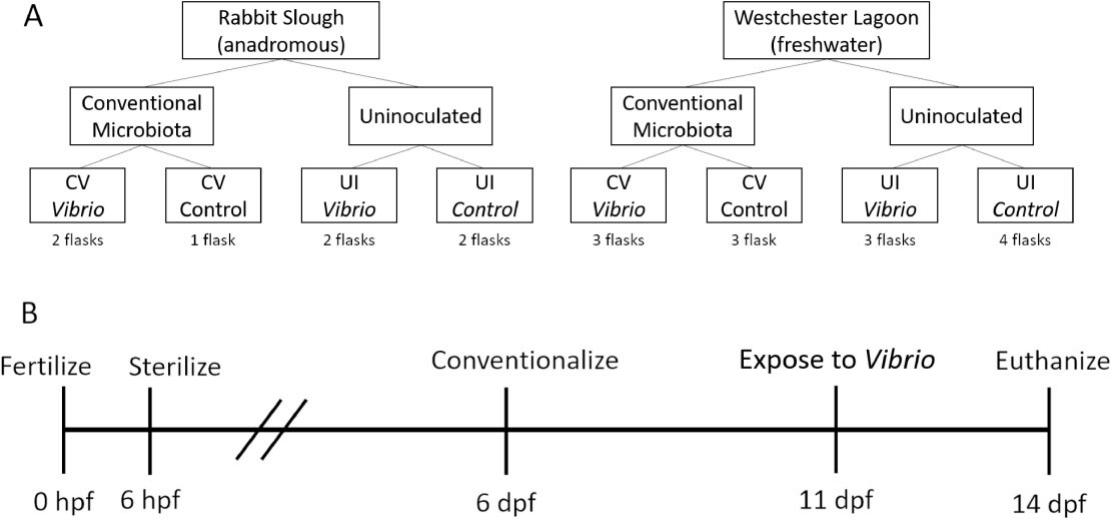
An overview of the full factorial design (**A**) and the timeline (**B**) of the experiment. Each flask contained 40 stickleback.

Here, we asked how the microbiota and host genetic background affects growth, development, and immunocompetence threespine stickleback. We tested the hypothesis that normal microbial colonization during early life promotes immunocompetence by upregulating host cytokine gene expression and affects growth and development. We further hypothesized that the genetic background of the host would affect the strength of immune responses, growth rate, and development. To these ends, we raised stickleback from two genetically divergent populations: one anadromous and one freshwater. We sterilized stickleback eggs and then exposed the larvae to environmental microbes or raised them without microbial inoculation. In the absence of reinoculation with conventional microbiota, this sterilization protocol affects microbiota community structure, immune function, metabolism, and morphological development, even if environmental microbes are allowed to begin recolonization immediately after treatment (Warne et al. 2019). Following microbial treatment, we exposed the stickleback to a potential fish pathogen, *Vibrio anguillarum*, in a full factorial design (Fig. 1). Although we could find no previous investigations explicitly testing the virulence of *V. anguillarum* in stickleback, Schade et al. (2014) found an unidentified species of *Vibrio* caused mortality in stickleback. Following our microbial treatments and *V. anguillarum* exposure, we quantified survival, growth, development, and immune gene expression from stickleback in each treatment.

## Methods

### Fish husbandry

Embryos were generated by *in vitro* crosses of wild-caught parental fish. Adult Rabbit Slough (61.53593, −149.2538°) and Westchester Lagoon resident freshwater (61.20616°, −149.92400°) were collected with one-fourth minnow traps set near shore and left overnight in June 2018 in accordance with the Alaska Department of Fish and Game permit P-18-006. Clutches from two females from Rabbit Slough and three females from Westchester Lagoon were fertilized *in vitro* in the field with the macerated testes from two males from the same population. Fertile eggs were sterilized the same day following the protocol used by Milligan-Myhre et al. (2016).

We incubated the eggs and macerated testes at room temperature for 2–4 h in 45 mm Petri dished with sterile 4 ppt instant ocean containing ampicillin (100 µg/mL), kanamycin (5 µg/mL), and amphotericin (250 ng/mL). We then transferred viable eggs to 100 mm Petri dishes and incubated them for a further 2 h at room temperature. Finally, we cleaned the remaining viable eggs with 0.2% polyvinylpyrrolidone-iodine, diluted in sterile 4 ppt instant ocean for 10 min, rinsed 3 times in sterile 4 ppt instant ocean (SBEM), soaked in 0.0003% bleach for 10 min, and rinsed another 3 times in sterile 4 ppt instant ocean.

Eight hundred sterilized eggs (520 from Westchester Lagoon and 280 from Rabbit Slough) were distributed into 20 sterile polystyrene flasks (40 eggs per flask) with filter caps (75 cm^2^, TPP Techno Plastic Products AG, Trasadingen, Switzerland) containing 50 mL of sterile 4 ppt instant ocean (Fig. 1). All protocols and procedures adhere to the University of Alaska Anchorage Institutional Animal Care and Use Committee (IACUC) approved methods for the ethical care and use of animals.

Sterility of water and embryos was assessed by direct visualization using phase optics at 40× magnification using an inverted microscope, and by culturing water aerobically on tryptic soy agar (TSA) plates at 18°C for 48 h. Observation of microbes on the bottom of the flask or in the water column or growth of colonies on the agar plates indicated contamination.

We also used the RNA that we isolated to test cytokine gene expression (see below) to test the germ-free status of the stickleback. Genomic DNA was removed from RNA samples using Invitrogen ezDNase kit according to the manufacturer’s protocol. RNA concentration was then measured using an Invitrogen Qubit version 4.0 fluorometer (Invitrogen, Waltham, MA, USA), and samples were made into working concentrations of 50 ng/μL. cDNA was generated from the working concentration RNA samples using the Invitrogen SuperScript IV First-Strand Synthesis System according to the manufacturer’s protocol (Invitrogen, Waltham, MA, USA).

cDNA was amplified using 10 μM 515 F and 806 R 16S primers (1 μL each/reaction) using GoTaq Master Mix (10 μL/reaction; Promega Corp., Madison, WI, USA), nuclease free water (12 μL/reaction), and 1 μL of template cDNA. Amplification was performed on an Applied Biosystems Veriti 96 Well Fast Thermal Cycler (Applied Biosystems, Foster City, CA, USA) with an initial 94°C for 3 min, 35 cycles of 94°C for 45 s, 50°C for 1 min, 72°C for 1 min 30 sec, followed by a final extension of 72°C for 10 min.

Immediately after 16S polymerase chain reaction (PCR) amplification samples and 5 μL of sample or 0′ Gene Ruler 1 kb+ Ladder (Fermentas, Waltham, MA, USA) were run on a 3% agarose 1X TAE gel at 90 V until bands were sufficiently separated. Bands were visualized under UV light. The presence of a band indicated the fish was colonized with bacteria. Lack of a band indicated the fish was not colonized.

### Confirmation of ability of *V. anguillarum* to colonize stickleback

Lab raised Rabbit Slough and Boot Lake embryos were generated by *in vitro* crosses. Fertile eggs from two clutches (one female, one male per clutch) were sterilized following the protocol used by Milligan-Myhre et al. (2016). Eggs were divided into three flasks and inoculated 10 days post fertilization with conventional water from tanks housing adult fish from the same family as the flask, 10^5.5^ CFU of KMM057, or left sterile. Sterility of the flasks was determined by visualization and plating on TSA plates left at 18°C for 48 h. At 14 days post fertilization (dpf), fish were euthanized according to an IACUC protocol approved by the University of Oregon, and guts from two fish per flask were aseptically dissected and homogenized in filter-sterilized SBEM, diluted, and plated in duplicate on TSA plates. Water was collected from each flask, diluted, and plated on TSA plates simultaneously. Plates were incubated at 18°C for 2 days prior to counting. All Rabbit Slough and Boot Lake fish were consistently colonized with between 10^2^ and 10^4^ CFU per mL. Water from all flasks contained >10^7^ CFU/mL.

### Microbial introductions

Half of the gnotobiotic flasks from each population were “conventionalized” at 6 dpf using 1 mL of water from fish tanks (Fig. 1). *Vibrio anguillarum* (strain KMM0057) were isolated from the intestine of an anadromous stickleback, collected from Millport Slough, OR, USA in 2010, and stored at −80°C following standard protocols and Milligan-Myhre et al. (2016). Bacteria were plated on TSA plates, incubated at 18°C for 48 h, and single colonies were inoculated into tryptic soy broth and incubated with shaking at 18°C until the culture was confluent. On 11 dpf, 10^5.5^ CFU of *V anguillarum* per mL was added to half of the flasks from each population (Fig. 1). Microbial conditions were confirmed at collection on 14 dpf by plating water on TSA plates and incubating at 18°C. We examined the plates for microbial growth at 48, 72, and 96 h.

### Tissue and image collection

Fish were euthanized at 14 dpf following IACUC approved protocols using a lethal dose of buffered MS222 (Fig. 1). Images were taken of each fish. We retained a subsample of three whole stickleback from each flask for immune gene analysis. Each whole body was placed into an individual RINO tube (Next Advance Inc., Troy, NY, USA) prefilled with 200 µL TRIzol and a mix of 0.5 and 1.0 mm zirconia oxide beads. RNA was isolated using a protocol modified from Leung and Dowling (2005) and Small et al. (2017). We thawed and bead-beat each sample on a Bead Mill24 homogenizer (Thermo Fisher Scientific, Waltham, MA, USA), added an additional 800 mL TRIzol, and flash froze the sample in liquid nitrogen again immediately afterward. We then spun the resulting homogenate through a Qiashredder centrifuge column (Qiagen, Hilden, Germany), performed two rounds of phase separation with chloroform in 2.0 phase-lock gel tubes (QuantBio, Beverly, MA, USA), and washed and eluted RNA using the RNeasy kit (Qiagen). We quantified the RNA using a Qubit 4 Fluorometer (Thermo Fisher Scientific, Waltham, MA, USA) and examined its integrity with an RNA IQ Asssay Kit (Thermo Fisher Scientific). All of the samples had an RNA IQ score >9.0, indicating the RNA had not degraded and consisted mainly of large molecules that maintained their tertiary structure. Finally, we assessed the purity of the RNA on a NanoDrop spectrophotometer (Model 840-274200, Thermo Fisher Scientific). All standards were standardized to 50 ng µL^−1^ with sterile, ultrapure water before immune gene quantification.

### Quantification of cytokine gene transcripts

We quantified cytokine gene expression using a one-step quantitative reverse transcription (RT-qPCR) (Luna Universal Probe One-Step RT-qPCR Kit, New England Biolabs, Ipswich, MA, USA) in triplicate 25 µL reactions and thermocyling that followed manufacturer’s recommendation. Each well contained 10 µL reaction mix, 1 µL WarmStart reverse transcriptase, 0.8 µL forward primer (10 µM), 0.8 µL reverse primer (10 µM), 6.4 µL ultrapure water, and 1 µL standardized RNA sample (50 ng µL^−1^). We assayed cytokines Interleukin 1β (IL-1β; Fwd-ACGGCTCGGAGTTGCTGAT, Rev-CTGCACAGCGTCACGATCTC) and tumor necrosis factor-α (TNF-α; Fwd-GCTTGGTTCTGGCCAGGTTT, Rev-GCTGCTGATTTGCCTCAACG), because they are measures of innate immunity that vary between stickleback populations (Robertson et al. 2016). We used two reference genes, beta-2-microglobulin (Fwd-AGACTATGCCTGGGAATCAAAC, Rev- GAAGATGTGTTGAATAGAAGCTGG), and L13A ribosomal binding protein (Fwd-CACCTTGGTCAACTTGAACAGTG, Rev- TCCCTCCGCCCTACGAC) identified by Hibbeler et al. (2008) as stable reference genes for stickleback. RNA was quantified on a BioRad CFX96 Touch Real-Time PCR Detection System (Bio-Rad Laboratories, Hercules, CA, USA). Following quantification, we normalized relative gene expression for IL-1β and TNF-α with the normPCR function in the SLqPCR package (Kohl 2007) in R version 3.5.3 (R Foundation for Statistical Computing).

### Image analysis

**Fig. 2:**
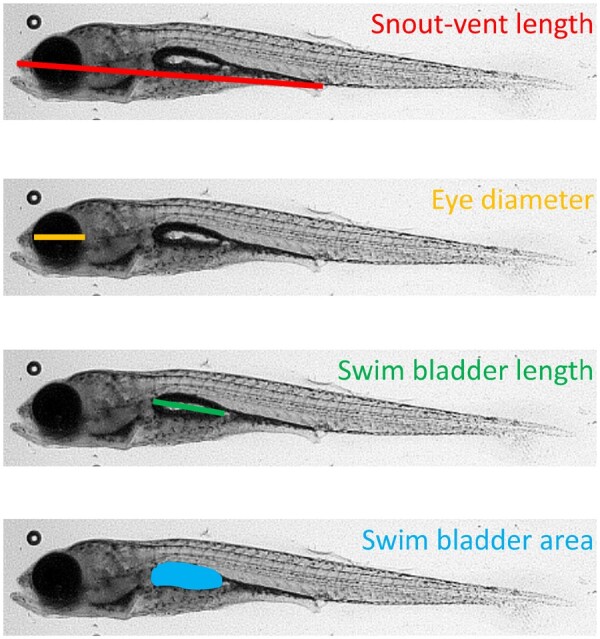
The morphological measurements of 14 dpf threespine stickleback.

Measurements were taken in triplicate using ImageJ (Schneider et al. 2012) and performed by the same researcher (L.K.). We measured snout–vent length (SVL), eye diameter, swim bladder length, and swim bladder area (Fig. 2). We chose SVL as a measurement of size, because, unlike total length, the position and bending of the tail do not affect SVL. We chose to measure eye diameter, swim bladder length, and swim bladder diameter because they are easily observed in 14 dpf stickleback and are useful for staging development in fish (Parichy et al. 2009). Swim bladder inflation (area) and swim bladder elongation (length), though correlated, represent different developmental processes (Ng et al. 2005; Robertson et al. 2007; Parichy et al. 2009).

### Statistical analysis

With respect to the recommendations of the American Statistical Association (Wasserstein et al. 2019), we have not set an alpha level for “significance.” We report our *P*-values as continuous variables and interpret the context of *P*-values in consideration of sample size and effect size. We used R version 4.0.1 (R Core Team 2020) for morphological and cytokine gene expression models and SAS version 9.4 (SAS Institute Inc.) for the survival model.

We used a mixed effects Cox regression to test stickleback survival. We included population, microbiota treatment, and *V. anguillarum* exposure as fixed effects in a full factorial design. We also included flask number as a random effect to account for variation between flasks.

We coded separate linear mixed effect models with the *lmer* function within the *lme4* package (Bates et al. 2019) to evaluate differences in size and morphology. We analyzed the size by coding SVL as a response variable and included population, microbiota treatment, and *V. anguillarum* exposure as fixed effects in a full factorial design. The morphology models used eye diameter, swim bladder length, and swim bladder area as response variables with population, microbiota treatment, and *V. anguillarum* exposure as fixed effects in a full factorial design, and SVL as a continuous covariate. All size and development models included flask number as a random effect to account for variation between flasks. We generated *P*-values using the *lmertest* package (Kuznetsova et al. 2017). We used Kenward-Roger Method for fixed effects and the *ranova* function for random effects.

Multivariate analysis of variance was used to analyze cytokine gene expression. We used the normalized relative expression output for IL-1β and TNF-α as response variables and population, microbiota treatment, and *V. anguillarum* exposure as fixed effects in a full factorial design. Flask number was included as an error term to account for variation between flasks. We examined the random effects of flasks with Bartlett tests.

## Results

### Microbial introductions

We did not observe any microorganisms via direct visualization in flasks that were uninoculated. Additionally, there was no visible growth on TSA plates from the water on either Days 2 or 14 in uninoculated flasks. To confirm whether the fish were germ free, we used universal primers to amplify the *16S* gene from RNA isolated from whole fish. While the flasks that were uninoculated showed no growth by visualization or growth on TSA plates, the PCR resulted in bands in some of the flasks (Supplementary Fig. S1). Primers used to amplify the *16S* gene are known to also amplify mitochondrial DNA (de la Cuesta-Zuluaga and Escobar 2016; Small et al. 2019), thus making the results of the 16S PCR unreliable. Furthermore, for two flasks, we did not have enough RNA to complete our analysis. Thus, we refer to the flasks as “uninoculated” rather than “germ free.” We could not conclude from the PCR that the fish in flasks that received *V. anguillarum* were colonized specifically by *V. anguillarum*. For this reason, we refer to the stickleback that was exposed to *V. anguillarum* as “exposed” rather than “colonized.”

### Survival

Microbiota treatments can affect the ability of hosts to survive in early stages of development. Specifically, *V. anguillarum* is a potential pathogen, and thus may have an effect on the survival of the host (Frans et al. 2011). To determine whether microbiota treatments differentially affected the populations in this study, we compared the number of eggs introduced to each flask at the start of the experiment to the surviving fish at the termination of the experiment.

The mixed effects Cox regression indicated differential survival (df = 14.4, *χ*^2^ = 41.3, *P* = 0.0002). This difference was most likely due to the random differences between flasks (df = 7.4, *χ*^2^ = 23.7, *P* = 0.002). None of the fixed effects, population (df = 1, *χ*^2^ = 0.41, *P* = 0.27), microbiota treatment (df = 1, *χ*^2^ = 0.53, *P* = 0.19), or *V. anguillarum* exposure (df = 1, *χ*^2^ = 0.85, *P* = 0.42) likely affected survival. It was similarly unlikely any interaction effect, population × microbiota treatment (df = 1, *χ*^2^ = 0.16, *P* = 0.26), population × *Vibrio* exposure (df = 1, *χ*^2^ = 0.18, *P* = 0.25), microbiota treatment × *V. anguillarum* exposure (df = 1, *χ*^2^ = 0.05, *P* = 0.44), or population × microbiota treatment × *V. anguillarum* exposure (df = 1, *χ*^2^ = 0.69, *P* = 0.16) affected survival. Thus, *V. Anguillarum* exposure did not affect survival of fish from these populations early in development.

### SVL

Microbiota can influence the growth of the host at various life stages. While we have insight to the contributions of microbes to overall development in some hosts, little is known about whether the host genetic background contributes to the ability of microbiota influence the overall growth of the host in the presence of *V. anguillarum*. Thus, we measured the SVL, a measurement of overall growth, in fish treated with various microbial communities.

The model for SVL did not show any effects of population (*F*_1, 11.79_ = 1.0, *P* = 0.32), microbiota treatment (*F*_1, 11.74_ = 3.5, *P* = 0.09), or *V. anguillarum* exposure (*F*_1, 11.75_ = 1.8, *P* = 0.2). SVL was not affected by first-order interactions population × microbiota treatment (*F*_1, 12.02_ = 0.1, *P* = 0.71), population × *V. anguillarum* exposure (*F*_1, 11.86_ = 0.1, *P* = 0.82), microbiota treatment × *V. anguillarum* exposure (*F*_1, 11.88_ = 0.1, *P* = 0.8), or the second-order interaction, population × microbiota treatment × *V. anguillarum* exposure (*F*_1, 12.15_ < 0.1, *P* = 0.87). Therefore, microbial treatment, whether with specific microbial members or complex communities, do not affect the early stages of growth in these populations. Flask identity accounted for 27% of the variation in SVL and likelihood ratio tests indicated this variance had an effect (*χ*^2^ = 65.4, *P* < 0.0001). However, visual analysis of each flask did not detect large deviances in the same direction from flasks in the same treatment (Supplementary Fig. S2).

### Eye diameter morphology

Microbes are able to interact with specific developmental pathways to influence individual pathways. Although we observed no gene by environment influences on overall growth, we wanted to determine whether the development of specific host organs was influenced by interactions between the host genetic background, microbial environment, or *V. anguillarum exposure*. To determine this, we measured relative sizes of the eye diameter, swim bladder length, and swim bladder volume compared to SVL.

**Fig. 3:**
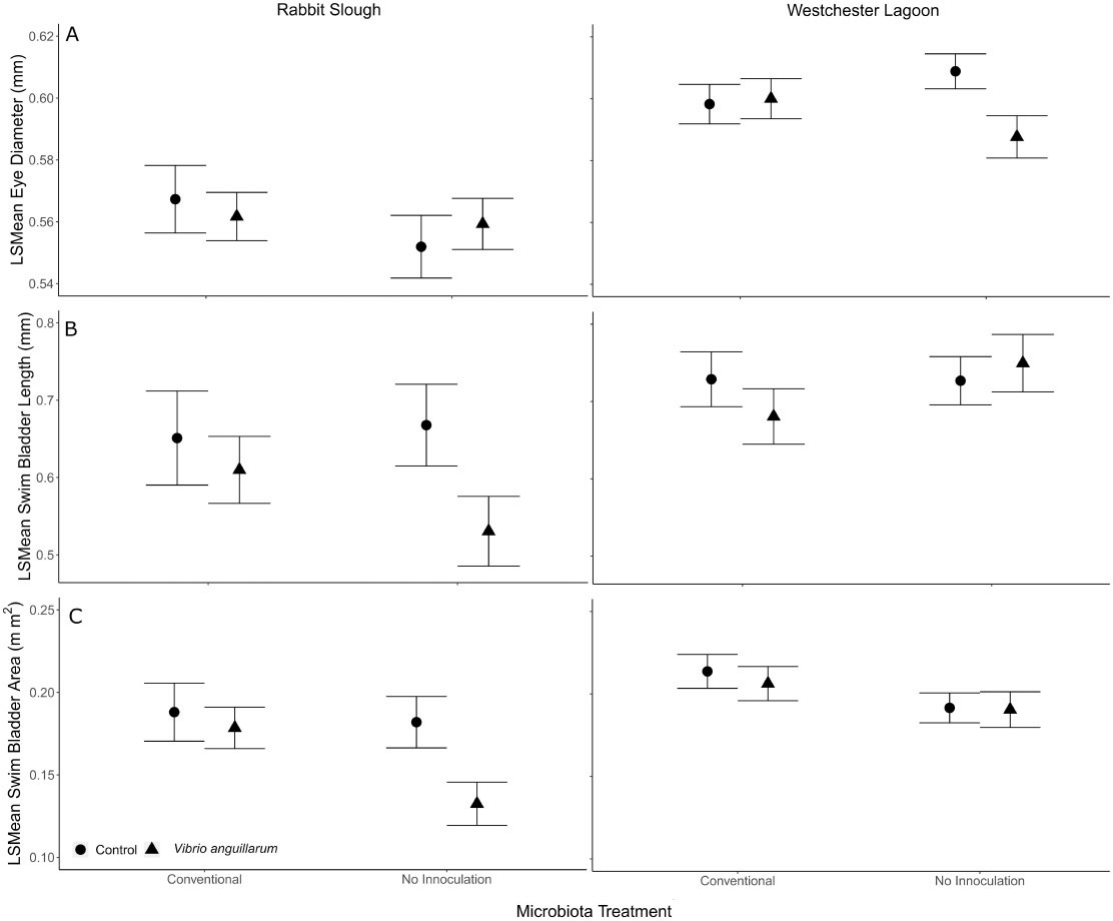
Least square means (±1 SE) of morphological measurements, eye diameter (**A**), swim bladder length (**B**), and swim bladder area (**C**), from anadromous (Rabbit Slough) and freshwater (Westchester Lagoon) stickleback by microbiota and *V. anguillarum* exposure. Least square means are from a linear mixed model with SVL as a covariate.

The overall developmental morphology model indicated that SVL had a strong relationship with eye diameter (*F*_1, 363.76_ = 527.7, *P* < 0.0001). Because of this strong effect, we report and graph least square means (±SE) from our model to account for eye diameter relative to body size. Overall, stickleback from Westchester Lagoon had longer eyes diameters, relative to their body size, than those from Rabbit Slough (Fig. 3A;*F*_1, 11.64_ = 43.2, *P* < 0.0001). Eye diameter was unaffected by the remaining fixed effects, microbiota treatment (*F*_1, 11.86_ = 0.1, *P* = 0.80), and *V. anguillarum* exposure (*F*_1, 11.57_ = 1.6, *P* = 0.23). Eye diameter was not affected by first-order interactions, microbiota treatment × *V. anguillarum* exposure (*F*_1, 11.72_ = 1.4, *P* = 0.25), microbiota treatment × population (*F*_1, 11.66_ = 0.5, *P* = 0.51), and population × *V. anguillarum* exposure (*F*_1, 12.14_ = 0.1, *P* = 0.71), or the second order interaction, population × microbiota treatment × *V. anguillarum* exposure (*F*_1, 12.53_ = 2.5, *P* = 0.13). Likelihood ratio tests indicated that flask identity had an effect on eye diameter (*χ*^2^ = 6.6, *P* = 0.01). However, visual analysis of the deviance of each flask did not detect large deviances in the same direction from flasks in the same treatment (Supplementary Fig. S3).

### Swim bladder morphology

The swim bladder in fish is analogous to the lung in mammals and complex developmental pathways are involved in the development of both (Yin et al. 2011; Zheng et al. 2011). To determine whether the ability of the microbiota and *V. anguillarum* exposure affect the development of these pathways depends on the host genetic background, we measured two aspects of swim bladder morphology, length, and volume.

SVL had a strong relationship with swim bladder length (*F*_1, 397.39_ = 313.4, *P* > 0.001). We report and graph least square means (±SE) from our model to account for swim bladder length relative to body size. Overall, stickleback from Westchester Lagoon tended to have longer swim bladders than those from Rabbit Slough (Fig. 3B;*F*_1, 11.68_ = 12.2, *P* = 0.005). Swim bladder length was unaffected by the remaining fixed effects, microbiota treatment (*F*_1, 11.83_ < 0.1, *P* = 0.88), and *V. anguillarum* exposure (*F*_1, 11.63_ = 1.3, *P* = 0.29). Swim bladder length was not affected by first-order interactions, microbiota treatment × *V. anguillarum* exposure (*F*_1, 11.74_ = 0.16, *P* = 0.70), microbiota treatment × population (*F*_1, 12.04_ = 1.8, *P* = 0.20), and population × *V. anguillarum* exposure (*F*_1, 11.69_ = 1.0, *P* = 0.33), or the second-order interaction, population × microbiota treatment × *V. anguillarum* exposure (*F*_1, 12.31_ = 1.8, *P* = 0.20). Likelihood ratio tests indicated that flask identity had an effect swim bladder length (*χ*^2^ = 18.0, *P* < 0.0001). This effect accounted for 12% of the overall variation of swim bladder length. Visual analysis of the deviance of each flask confirms the small effect (Supplementary Fig. S4).

The overall developmental morphology model indicated that SVL had a strong relationship with swim bladder area (*F*_1, 389.1_ = 325.3, *P* < 0.0001) and we report and graph least square means (±SE) from our model in order to account for swim bladder area relative to body size. Stickleback from Westchester Lagoon tended to have larger swim bladder areas than those from Rabbit Slough (Fig. 3C;*F*_1, 11.66_ = 12.6, *P* = 0.004). The model also indicated an effect of microbiota treatment (*F*_1, 11.84_ = 8.1, *P* = 0.02). Uninoculated stickleback had smaller swim bladder areas (Fig. 3C). Swim bladder area was unaffected by *V. anguillarum* exposure (*F*_1, 11.61_ = 1.5, *P* = 0.24). Swim bladder area was unaffected by first order interactions, population × *V. anguillarum* exposure (*F*_1, 11.68_ = 1.4, *P* = 0.26), microbiota × *V. anguillarum* exposure (*F*_1, 11.73_ = 0.17, *P* = 0.68), population × microbiota (*F*_1, 12.06_ = 0.46, *P* = 0.51), or the second-order interaction, population × microbiota × *V. anguillarum* exposure (*F*_1, 12.37_ = 1.7, *P* = 0.22) did not seem to affect swim bladder area. Likelihood ratio tests indicated that flask identity had an effect swim bladder area (*χ*^2^ = 14.6, *P* = 0.0001). This effect accounted for 10% of the overall variation of swim bladder length. However, visual analysis of the deviance of each flask did not detect large deviances in the same direction from flasks in the same treatment (Supplementary Fig. S5) and we do not believe this is responsible for the differences in swim bladder area between microbiota treatments.

#### Cytokine gene expression

Resource allocation during development has been hypothesized to contribute to either development or immune response. Our previous work showed neutrophil abundance in intestines depended on host genetic background and microbial exposure (Milligan-Myhre et al. 2016). To determine whether the differences we observed in development is reflected in development of the immune response, we measured transcripts involved in cytokine signaling.

**Fig. 4:**
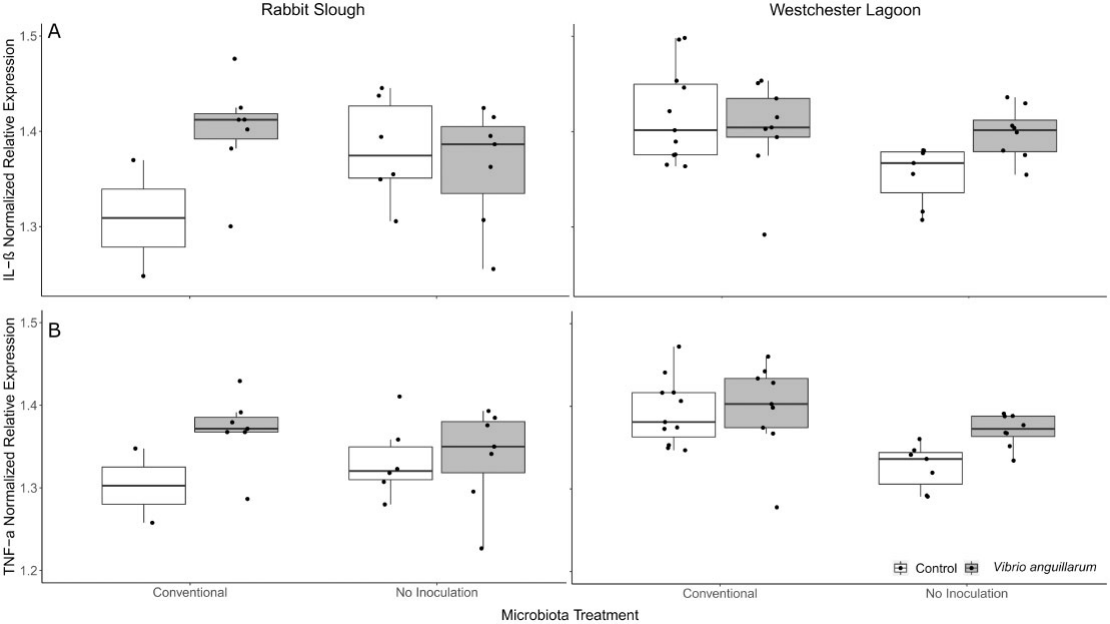
Normalized relative expression of cytokine genes IL-β (**A**) and TNF-α (**B**) from anadromous (Rabbit Slough) and freshwater (Westchester Lagoon) stickleback by microbiota and *V. anguillarum* exposure. Lines inside boxes indicate medians, boxes indicate 25th and 75th percentiles, bars indicate 1.5 × interquartile range, and points are individual measurements.

The model indicated that stickleback with conventional microbiota had higher levels of relative IL-1β (Fig. 4A;*F*_1, 12_ = 5.6, *P* = 0.04) and TNF-α expression (Fig. 4B;*F*_1, 12_ = 13.4, *P* = 0.003) than uninoculated stickleback. Stickleback from Westchester Lagoon had slightly higher relative TNF-α expression than those from Rabbit Slough (Fig. 4B;*F*_1, 12_ = 5.5, *P* = 0.04), but the IL-1β expression did not differ between the populations (Fig. 4A;*F*_1, 12_ = 2.2, *P* = 0.16). The model also indicated an effect of the population × microbiota × *V. anguillarum* exposure interaction on relative IL-1β expression (*F*_1, 12_ = 11.3, *P* = 0.006). Stickleback from Rabbit Slough with conventional microbiota had higher relative expression of both IL-1β and TNF-α when exposed to *V. anguillarum* (Fig. 4). Uninoculated stickleback from Westchester Lagoon had higher relative IL-1β when exposed to *V. anguillarum* (Fig. 4A). The same pattern is evident in TNF-α population × microbiota × *V. anguillarum* but has less support in the model (Fig. 4B;*F*_1, 12_ = 4.3, *P* = 0.06). Overall, these results should be interpreted with caution as the effect sizes are relatively small and the flask number for each treatment is low when considering the population × microbiota × *V. anguillarum* interaction, especially from Rabbit Slough (Fig. 1).

IL-1β was not affected by infection (*F*_1, 12_ = 1.0, *P* = 0.35) or any first-order interactions, microbiota treatment × *V. anguillarum* exposure (*F*_1, 12_ < 0.1, *P* = 0.86), microbiota treatment × population (*F*_1, 14_ = 1.4, *P* = 0.25), and population × *V. anguillarum* exposure (*F*_1, 12_ = 1.0, *P* = 0.77). Bartlett tests did not indicate a random effect of flask (*K*^2^ = 13.1, df = 18, *P* = 0.83).

TNF-α was not affected by infection (*F*_1, 12_ = 3.4, *P* = 0.8) or any first-order interactions, microbiota treatment × *V. anguillarum* exposure (*F*_1, 12_ = 0.1, *P* = 0.78), microbiota treatment × population (*F*_1, 14_ = 2.1, *P* = 0.17), and population × *V. anguillarum* exposure (*F*_1, 12_ < 0.1, *P* = 0.92). Bartlett tests did not indicate a random effect of flask (*K*^2^ = 22.4, df = 18, *P* = 0.27).

## Discussion

Our results indicate disruption of the microbiota may affect organ development in early life. However, this effect was trait specific. Stickleback that was inoculated with conventional microbiota had larger swim bladder areas relative to those that remained uninoculated, but eye diameter remained unaffected. Exposure to a potential pathogen, *V*. *anguillarum*, may have affected cytokine gene expression, but did not affect survival.

Animals raised with radically altered microbiota can exhibit abnormal development (Shin et al. 2011; Sommer et al. 2016; Warne et al. 2019). In our study, uninoculated stickleback had smaller swim bladders than those raised with conventional microbiota. In teleost fish, the swim bladder forms from, and remains connected to, the foregut (Robertson et al. 2007). The appearance of the swim bladder in stickleback coincides with an elongation of the intestines (Swarup 1958). It is possible that microbial colonization or signaling may play a role in swim bladder development in teleost fish. This is consistent with other investigations that have found morphological changes in tissues beyond the gut associated with altered microbiota (Sommer and Bäckhed 2013). Tadpoles exposed to the same sterilization protocol we used, uninoculated with conventional microbiota, and colonized by environmental microbes exhibited radically-altered gut biota and spinal deformities that may be due to deficiencies of micronutrients ordinarily synthesized or metabolized by their normal microbiota (Warne et al. 2019). Similarly, germ-free mice have higher bone density because they have significantly lower levels of serotonin, which is synthesized by the microbiota and inhibits bone mineralization (Sommer and Bäckhed 2013). Investigations of the effects of direct microbial colonization and indirect microbial metabolites on swim bladder development may help explain the role of the microbiota in human health and development. Fish swim bladders and human lungs share molecular homologies (Zheng et al. 2011) and develop using the same signaling pathways (Yin et al. 2011). Metabolites that originate from the maternal microbiota could affect these signaling pathways (Macpherson et al. 2017) and there is evidence of microbial colonization of fetuses *in utero* (Younge et al. 2019). In this light, it is important to reiterate that the alterations in swim bladder development were likely due to early-life disruption of the microbiota in uninoculated stickleback, and not true germ-free conditions.

Microbial colonization in early life affects the development the host immune system (Kanther and Rawls 2010; Hooper et al. 2012; Sommer and Bäckhed 2013; Gensollen et al. 2016; Thaiss et al. 2016; McCoy et al. 2017) and host genetic background can also moderate the immune response to the microbiota (Milligan-Myhre et al. 2016; Small et al. 2017). We found differences in cytokine gene expression between microbiota treatments (Fig. 4). While the effects were small, they give possible direction for future research as there are physiological mechanisms that can explain the trends we found. Uninoculated stickleback had less relative expression of IL-1β and TNF-α. Conventional microbiota can upregulate pro-IL-1β, the inactive precursor to IL-1β and trigger intestinal macrophages to produce IL-1β (Kamada et al. 2013; Thaiss et al. 2016). A healthy microbiota is also associated with upregulation of TNF expression (Kamada et al. 2013). However, the dysbiosis associated with obesity can result in chronic, low-level, systemic inflammation, due, in part, to upregulation of TNF-α (Conterno et al. 2011; Clemente et al. 2012; Hooper et al. 2012; Tremaroli and Bäckhed 2012). We also found weak evidence that host genetic background affected cytokine gene expression. However, unlike previous studies which found stronger inflammatory responses and gene upregulation in anadromous populations (Milligan-Myhre et al. 2016; Small et al. 2017), we found higher relative TNF-α in our freshwater population from Westchester Lagoon. This highlights the need for more investigations on how adaptation to local environment hosts immune responses to the microbiota.

There were no differences in mortality between control stickleback and those exposed to *V. anguillarum*. This may have occurred because our strain was nonpathogenic in stickleback because the time of exposure time was too brief, or because our inoculum size was too small. *Vibrio anguillarum* is a usual member of the free-living, aquatic microbial community and has been isolated from multiple species of fish (Austin and Austin 2012). Two serotypes of *V. anguillarum* are responsible for mass die-offs of wild and farmed fish, while others have been isolated from marine water, sediment, and healthy fish (Toranzo and Barja 1990; Pedersen et al. 1999; Frans et al. 2011; Austin and Austin 2012). However, many of the strains isolated from environmental sources and healthy fish have iron-uptake mechanisms similar to pathogenic strains (Lemos et al. 1991). *Vibrio anguillarum* present in the intestinal microbiota of healthy fish can cause massive mortality when it becomes the dominant member of the microbiota (Grisez et al. 1997) and serotypes other than the two typically associated with mass die-offs can be pathogenic in fish (Larsen et al. 1994; Pedersen et al. 1999).

Larval fish have immature immune systems that lack an adaptive response and their intestinal pH is too high to inhibit *V. anguillarum* colonization and growth (Pedersen et al. 1999; Frans et al. 2011; Uribe et al. 2011; Vadstein et al. 2013; Gradil et al. 2014). Although colonizing microbiota can offer some degree of protection from pathogens via exploitative competition and competitive exclusion (Gómez and Balcázar 2008; Arrieta et al. 2014; Thaiss et al. 2016). For example, probiotic supplementation of *Roseobacter* can significantly reduce *V. anguillarum* mortality in larval fish (Planas et al. 2006). The age of our animals may account for the patterns of cytokine gene expression. Overall, stickleback exposed to *V. anguillarum* did not upregulate their cytokine gene expression, possibly because *V. anguillarum* can inhibit the inflammatory response in fish. Sea bass (*Dicentrarchus labrax*) injected with *V. anguillarum* exhibited only minimal increases in TNF-α expression and no increase in IL-1β (Sepulcre et al. 2007). However, we observed a potential pattern in which host genetic background and the microbiota seemed to affect the host immune response. Stickleback from Rabbit Slough raised with conventional microbiota and uninoculated stickleback from Westchester Lagoon may have had stronger expression of cytokine genes when exposed to *V. anguillarum*. Future studies should attempt to replicate this pattern with a larger sample size and perhaps more populations of stickleback.

In other fish without conventional microbiota, random tank effects influence both growth and susceptibility to pathogens. In salmon, a nongenetic tank effect played a small but not insignificant role in susceptibility to the infectious pancreatic necrosis virus for one family but not a second (Kjøglum et al. 2005). In other studies, random tank effects influenced growth in salmon (Herbinger et al. 1999) and trout (Speare et al. 1999). Given our current knowledge about how the microbiota can vary from one tank to another and the role of microbiota in development, it would be interesting to determine whether the microbiota varied from tank to tank in a similar experiment. In wild, adult stickleback microbiota composition is more strongly associated with genetic background than environment or food (Smith et al. 2015; Steury et al. 2019). Furthermore, there is evidence that genetic background of larval stickleback affects the translocation of immune cells to the gut in the presence or absence of microbiota (Milligan-Myhre et al. 2016). Despite these differences, *Pseudomonas*, *Chromobacterium*, *Shewanella*, and *Plesiomonas* have been found in lab-raised and wild stickleback from both anadromous and freshwater populations. These taxa typically colonize the GI tracts of many fish species (Nayak 2010). However, it remains unclear what role they may play in stickleback immunity or development. We also do not know whether the immature immune systems of larval fish can exert the same selective pressures on colonizing microbiota as the immune systems of adult fish. In this study, the random flask assignment affected all of our morphological parameters and survival, but not immune gene response. Despite these random effects, the microbiota effects did seem to drive specific developmental processes. To our knowledge, there is no information on the influence of the adaptive immune system in early-stage stickleback. However, given that zebrafish do not have a productive adaptive immune response until they are well past their juvenile stage (Lam et al. 2004), stickleback likely follow a similar developmental trajectory. Early microbial communities in zebrafish are stochastic, indicating a random, neutral colonization process in the juvenile stage (Burns et al. 2016). Thus, the conventionally raised juvenile stickleback in this experiment likely were colonized by a random microbial community, preventing a study of the Anna Karenina effects of microbial disruption in pathogen exposed fish (Zaneveld et al. 2017).

In conclusion, we showed that disrupting the microbiota in early life can affect the development of some host organs and possibly cytokine gene expression. This has implications for the direction of evolution on host–microbe interactions and human health. While this study does not explore long term effects of either microbial disruption, it does lay the groundwork for important developmental experiments in hosts with similar but distinct genetic backgrounds.

## Supplementary Material

icaa136_Supplementary_Data

## Notes

From the symposium “SICB Wide Symposium: Building Bridges from Genome to Phenome: Molecules, Methods and Models” presented at the annual meeting of the Society for Integrative and Comparative Biology, January 3–7, 2020 at Austin, Texas.
