# Supplementary material for: Early Life Disruption of the Microbiota Affects Organ Development and Cytokine Gene Expression in Threespine Stickleback
Source: Integr Comp Biol. 2020 Sep 24;63(1):250–62. doi: 10.1093/icb/icaa136 (PMC10388389; doi:10.1093/icb/icaa136)
Supplement: icaa136_Supplementary_Data [file icaa136_supplementary_data.zip › icb-2020-0012-File006.docx]

Supplemental figure 1. Confirmation of inoculation status. The 16S gene was amplified from RNA isolated from whole fish. + in the top line indicates whether the flask received conventional microbes. + in the bottom line indicates whether the flask received *Vibrio*. Each lane represents a single fish. Flask refers to the flask identification.

Figure S2. Deviance of snout-vent length, the response variable in a linear mixed model, as a random effect of flask ID.

Figure S3. Deviance of eye diameter, the response variable in a linear mixed model, as a random effect of flask ID.

Figure S4. Deviance of swim bladder length, the response variable in a linear mixed model, as a random effect of flask ID.

Figure S5. Deviance of swim bladder area, the response variable in a linear mixed model, as a random effect of flask ID.
