# Supplementary figures and images for: Early Life Disruption of the Microbiota Affects Organ Development and Cytokine Gene Expression in Threespine Stickleback
Source: Integr Comp Biol. 2020 Sep 24;63(1):250–62. doi: 10.1093/icb/icaa136 (PMC10388389; doi:10.1093/icb/icaa136)

|         |   |   |   |   |   |   |   |   |   |   |   |   |   |   |   |   |   |   |   |   |   |   |  |
|---------|---|---|---|---|---|---|---|---|---|---|---|---|---|---|---|---|---|---|---|---|---|---|--|
| Microbe | L | + | - | + | + | + | + | + | + | + | - | - | - | - | - | - | - | - | - | - | - | L |  |
| Vibrio  |   |   |   |   |   |   |   |   |   |   | + | + | + | + | + | + | - | - | - | - | - |   |  |
| Flask   |   |   |   | Q | Q | Q | Q | Q | Q | M | S | A | A | B | B | E | I | I | M | O | O | R |  |

1500 kb

500 kb

400 kb

300 kb

200 kb

75 kb

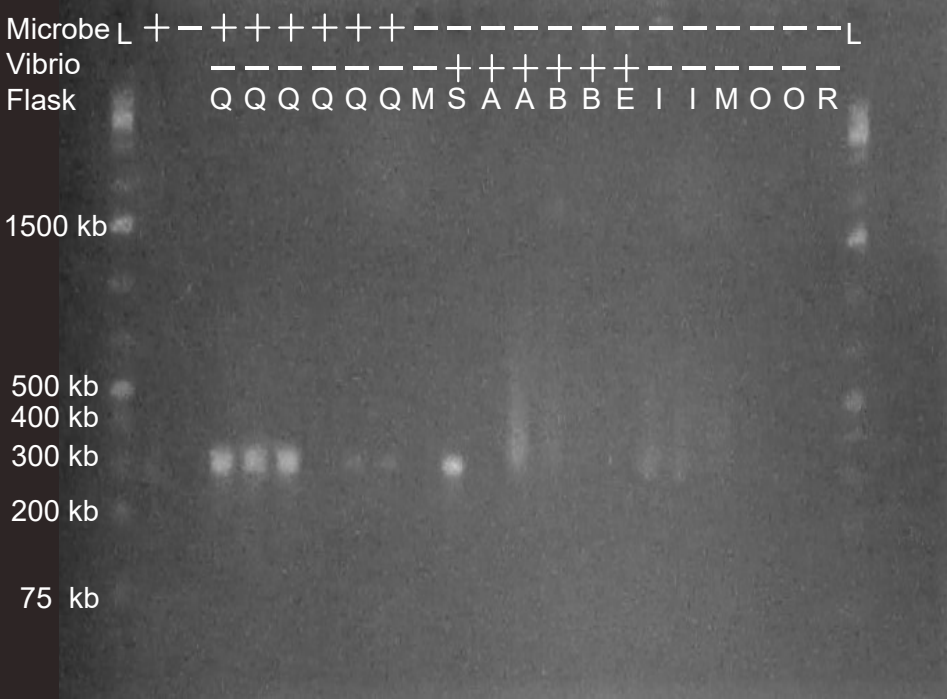

Supplement: icaa136_Supplementary_Data [file icaa136_supplementary_data.zip › icb-2020-0012-File007.pdf]

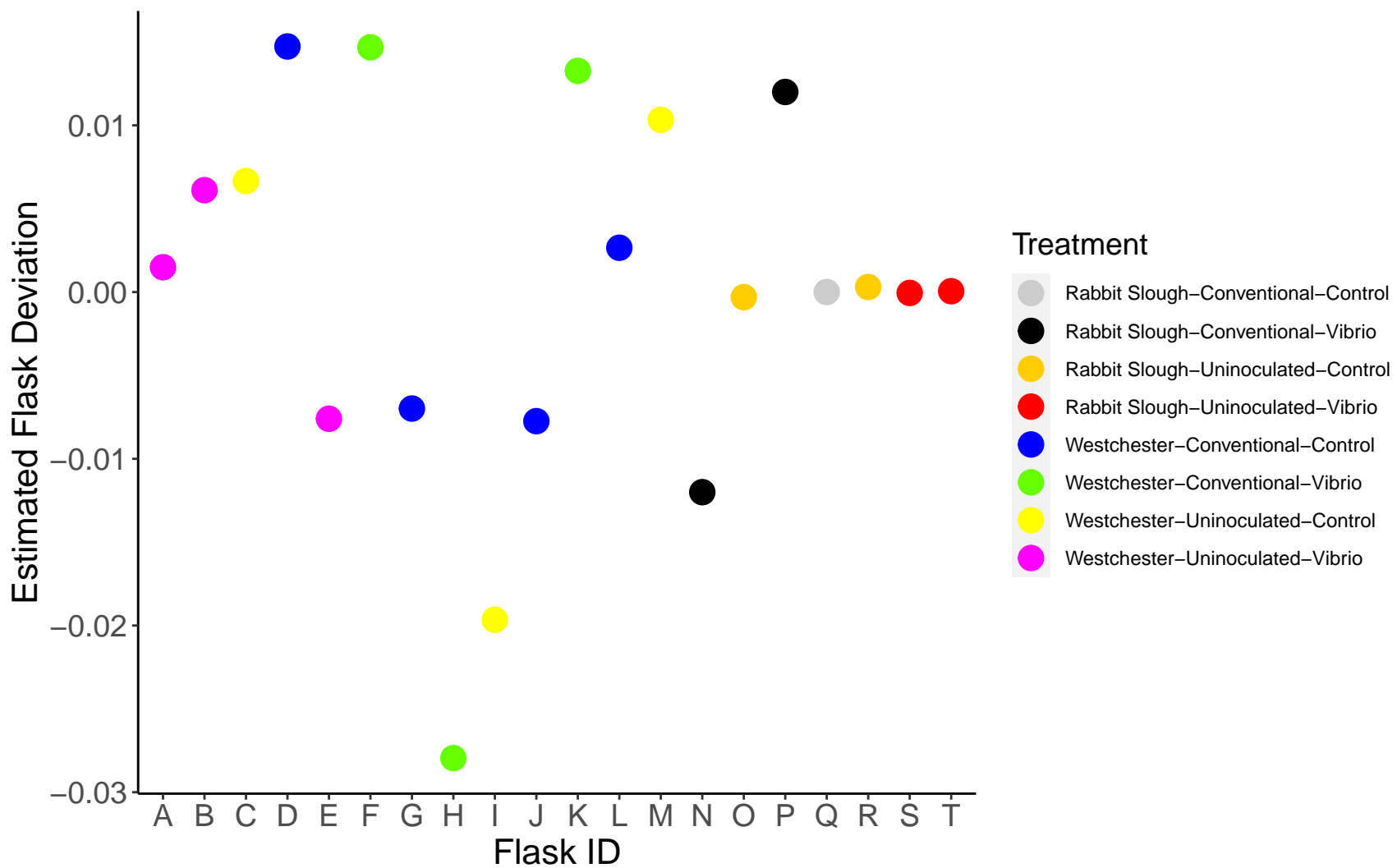

Supplement: icaa136_Supplementary_Data [file icaa136_supplementary_data.zip › icb-2020-0012-File008.pdf]

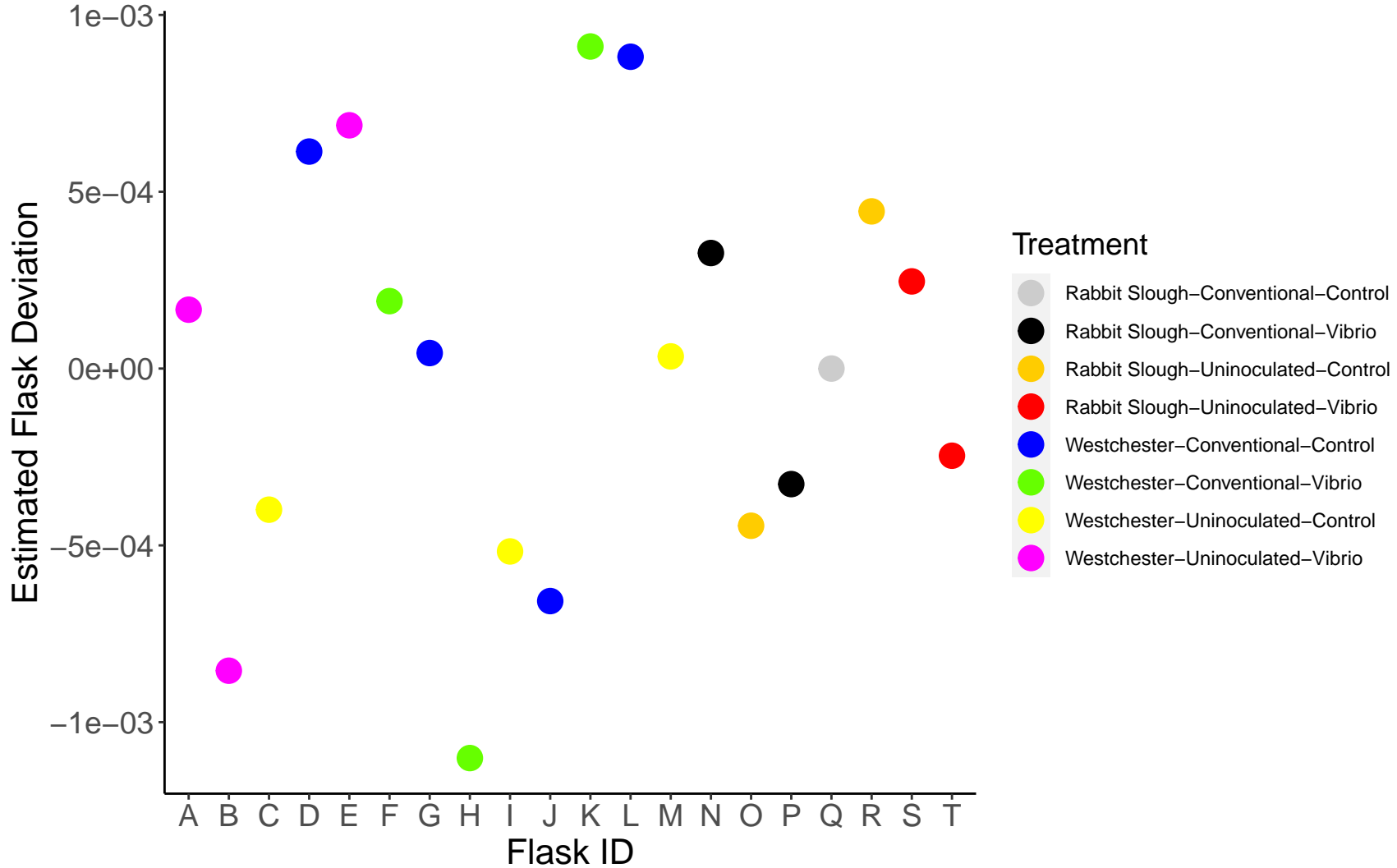

Supplement: icaa136_Supplementary_Data [file icaa136_supplementary_data.zip › icb-2020-0012-File009.pdf]

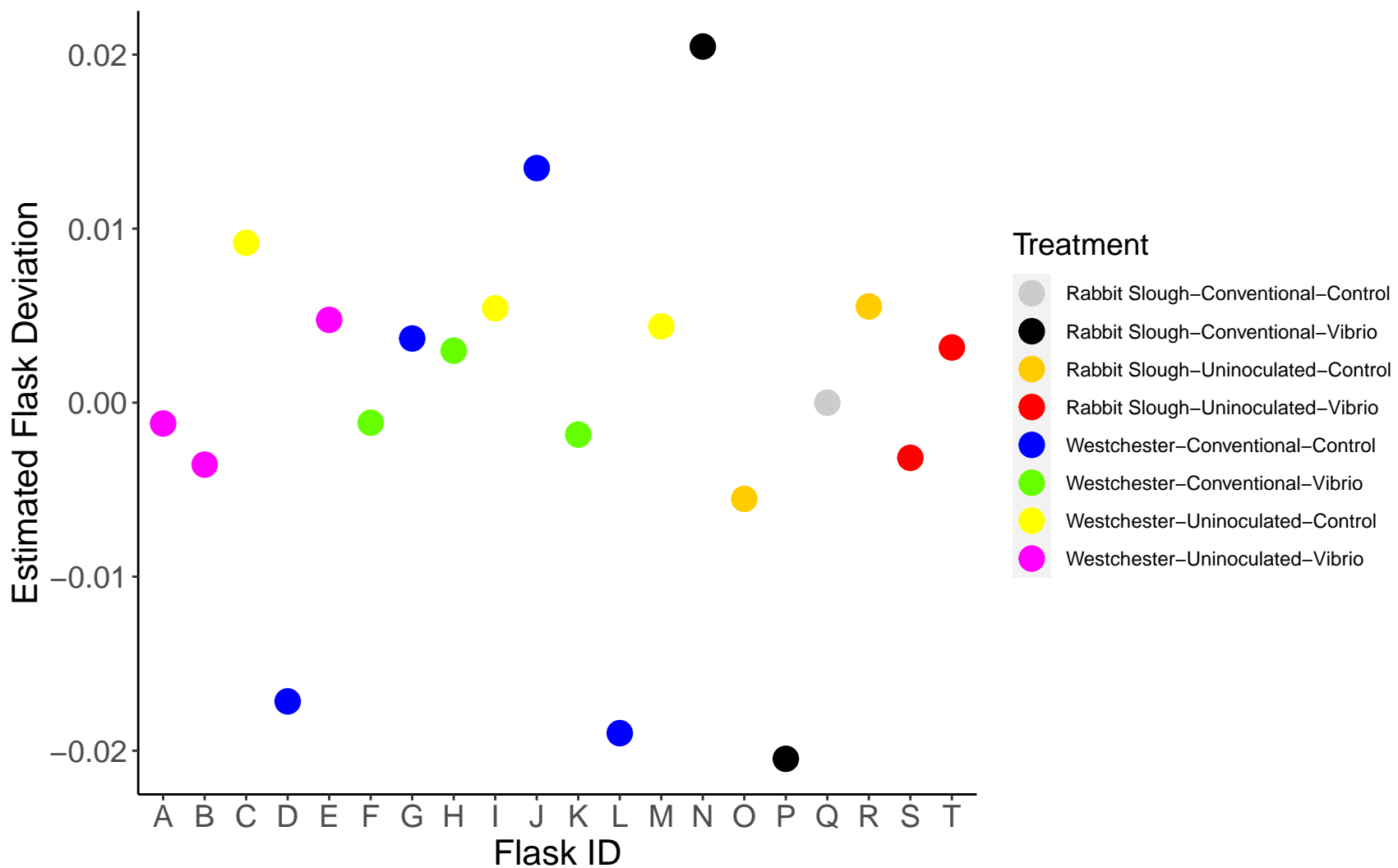

Supplement: icaa136_Supplementary_Data [file icaa136_supplementary_data.zip › icb-2020-0012-File010.pdf]

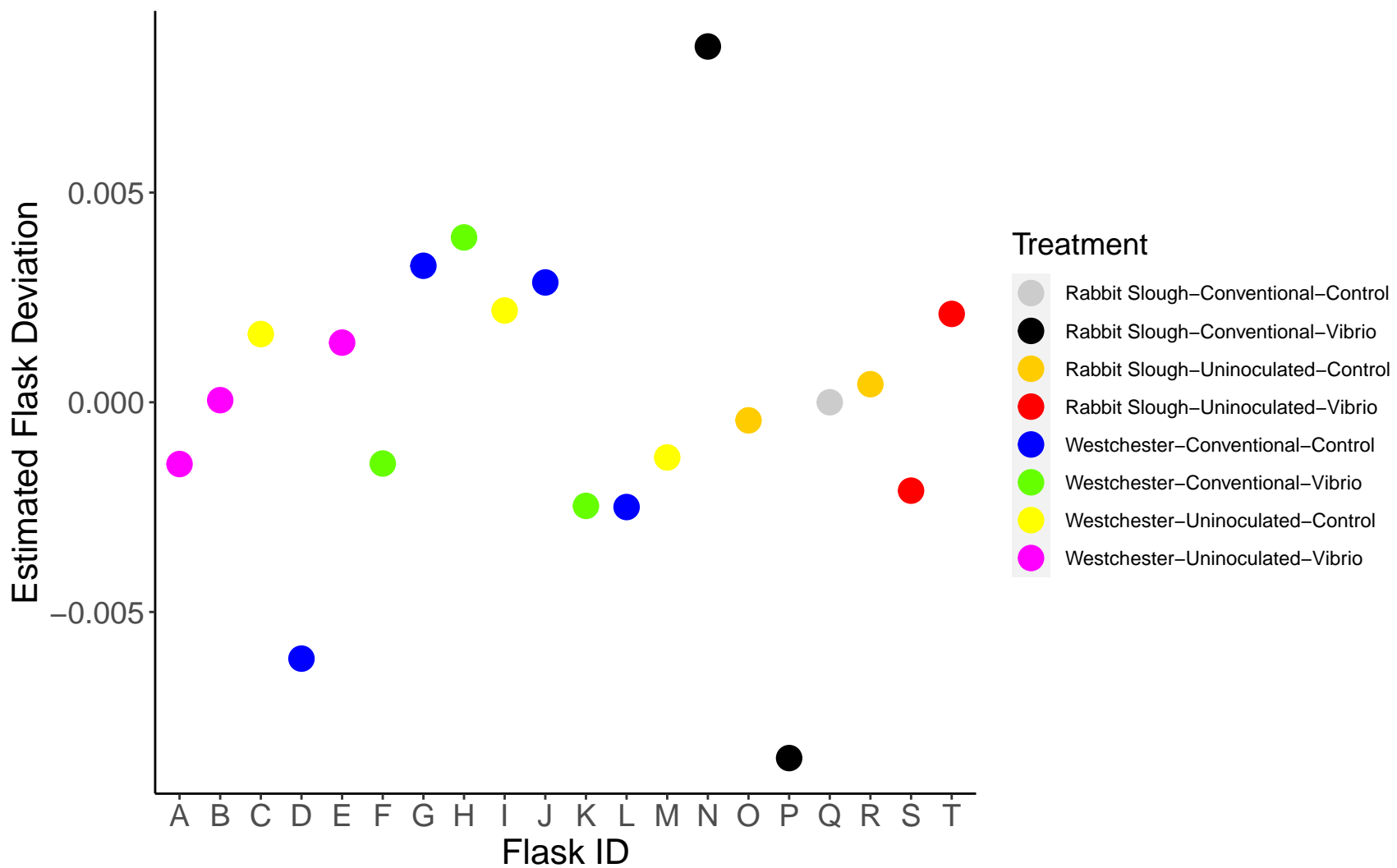

Supplement: icaa136_Supplementary_Data [file icaa136_supplementary_data.zip › icb-2020-0012-File011.pdf]
